# A Scoping Review of Preclinical Research on Monoclonal Antibody Development for Prophylaxis and Treatment of West Nile Virus Infections

**DOI:** 10.3390/v17060845

**Published:** 2025-06-12

**Authors:** Amanda E. Calvert, Kerri L. Miazgowicz, Bailey Atkinson, Audrey H. Long, Elisa Thrasher, Aaron C. Brault, Randall J. Nett

**Affiliations:** Arboviral Diseases Branch, Division of Vector-Borne Diseases, U.S. Centers for Disease Control and Prevention, Fort Collins, CO 80521, USA; ncd0@cdc.gov (K.L.M.); atkin353@alumni.umn.edu (B.A.); ktp4@cdc.gov (A.H.L.); elisa.thrasher@gmail.com (E.T.); zlu5@cdc.gov (A.C.B.); gge5@cdc.gov (R.J.N.)

**Keywords:** West Nile virus, flavivirus, monoclonal antibody, antibody therapeutics

## Abstract

West Nile virus (WNV) causes thousands of arboviral infections in the United States each year. Patients with immune-compromising conditions and elderly people are at higher risk of severe WNV neuroinvasive disease (WNND). Despite its broad endemicity nationwide, no U.S. Food and Drug Administration-approved vaccine or therapeutic treatments exist. We summarized existing peer-reviewed literature on the preclinical development of monoclonal antibody (MAb) prophylaxis and therapeutics for the prevention and treatment of WNND. Five bibliographical databases (CINAHL, Cochrane Library, Embase, MEDLINE, and Scopus) were searched for applicable research studies performed from 1 January 1998 to 1 May 2025. In total, 2347 titles and abstracts were screened, 263 full-text publications reviewed, and 25 studies included. Studies included detailed preclinical development and evaluations of MAbs targeting the envelope (E) protein (*n* = 13), other viral proteins (*n* = 3), flaviviral cross-protective monoclonal antibodies (*n* = 4), and novel antibody configurations or delivery methods (*n* = 5). The most well-studied MAb, E16, targeting E- Domain III (E-DIII), was effective at inhibiting and treating WNND in experimental animal models. No work investigated ways to traffic therapeutic antibodies across the blood–brain barrier. This review summarizes the current research in the development of monoclonal antibody therapeutics for WNV and addresses gaps in the knowledge for future consideration.

## 1. Introduction

West Nile virus (WNV) was first isolated in Uganda in 1937 and is endemic in many parts of the world including Africa, Europe, Asia, Australia, the Middle East, and North America [1]. Its first identification in the Western Hemisphere in 1999 was marked by rapid geographic expansion across the country in the years that followed. WNV is the leading cause of mosquito-borne human encephalitis in the United States, and has caused more than 30,000 cases of human WNV neuroinvasive disease (WNND) and more than 2800 deaths [2]. WNV is estimated to have caused nearly 7 million infections since its introduction to the United States, with an estimated annual healthcare cost of USD 56 million based on data accumulated from 1999 to 2012 [3]. For individual patients, healthcare costs can differ depending on clinical syndromes, including acute flaccid paralysis (median USD 25,117) and encephalitis (median USD 20,105) [3].

Populations disproportionately affected by WNV disease include people experiencing homelessness, elderly individuals, and people with immunocompromising health conditions or those taking medications that weaken their immune system (i.e., B-cell-depleting immunotherapies) [4]. Patients with WNND who are immunocompromised and aged ≥65 years are at higher risk for poorer outcomes and higher mortality compared with immunocompetent patients of similar age [5]. According to the 2020 census, an estimated 55,792,501 persons aged ≥65 years live in the United States [6]. Further, a recent analysis demonstrated that 2.8% of adults aged 18–64 years who had continuous commercial medical insurance coverage during 1 January 2017–31 December 2019 had drug-induced immunosuppression in a 365-day period [7]. Applying this finding to 2020 U.S. Census data reveals that approximately 5,761,442 U.S. adults likely had drug-induced immunosuppression during a 365-day period. When combining those aged at least 65 years with those who are immunosuppressed, an estimated 61,500,000 U.S. residents have an elevated risk of severe arboviral disease during the transmission season.

Currently, no Federal Drug Administration (FDA)-approved vaccines exist to protect against WNV infections. Only 10 clinical trials have been conducted to evaluate seven vaccine candidates, and none of these candidates have progressed past Phase II [4], likely due to an inability to predict WNV disease outbreaks in a given geographic area from year to year, making enrollment in a large efficacy study unfeasible [8]. Safety concerns with live-attenuated virus vaccine candidates also exist, as the target population (persons > 50 years or with immunocompromised conditions) that would most benefit from a WNV vaccine would also likely be more susceptible to serious vaccine-adverse events [8]. A WNV vaccine program would need to include an age- and incidence-based strategy to be most cost-effective and successful [9].

No FDA-approved therapies for WNND currently exist. WNND treatment is limited to supportive care and broad-spectrum supplemental therapies such as intravenous immunoglobulin (IVIG) and interferon alpha (IFNα) [5]. Monoclonal antibodies (MAbs) are promising drugs for use as prophylaxis and therapeutics to protect patients at risk of severe WNV disease. MAbs have become important tools as therapeutic agents for a variety of infectious and non-infectious diseases including cancer and autoimmune diseases, with more than 100 antibody drugs authorized for use by the FDA in 2021 [10]. MAbs provide alternatives for prophylactic intervention compared with traditional vaccines for persons unable to receive vaccination based on immune status, or antivirals and therapeutics for those patients presenting with serious illness.

This scoping review summarizes the literature on the preclinical development of MAbs for the prevention or treatment of severe WNV disease. We began our initial analysis including all preclinical studies of antivirals developed for the prophylaxis or treatment of WNV. Following the initial analysis, we narrowed the scope to MAb candidates. Specifically, we asked the following questions: What animal models have been developed for preclinical efficacy studies of WNV MAb therapeutics? What are the viral targets of these MAbs? Have novel delivery platforms been investigated for WNV therapeutics? Our analysis revealed key gaps in research, including a lack of studies conducted on engineering MAbs or developing delivery platforms to circumvent the blood–brain barrier (BBB), a lack of studies evaluating combination therapies to multiple WNV antigen targets to reduce the risk of viral escape, and a lack of practical therapies that take into account limitations in conducting large-scale human clinical trials that include an inability to predict WNV transmission or detect acute infections.

## 2. Materials and Methods

Overview. We conducted a literature search using the following keywords: West Nile virus, important components (envelope (E) protein, nonstructural protein 1 (NS1), nonstructural protein 3 (NS3), nonstructural protein 5 (NS5), host factors), antivirals (immunoglobulin, monoclonal antibody, viral inhibitors), prophylaxis, therapeutic. We aimed to include studies on modes of delivery, including transport compounds, alternative routes, and methods to bypass the blood–brain barrier. The search only included animal models tested for preclinical trials including rodents, non-human primates, and horses. The manuscript follows the checklist outlined in PRISMA for scoping reviews (PRISMA-ScR) [11].

Search strategy and selection criteria. The following databases were used to identify relevant studies: Medline, Embase, Cochrane library, CINAHL, and Scopus. The search included papers accepted in peer-reviewed journals written in English between 1 January 1998 and 16 May 2025. No grey literature was included in the search. The search strategy was conducted by an experienced librarian. Title and abstract reviews were completed in Covidence and conducted by 4 reviewers working in random pairs (K.M., A.L., B.A., and E.T.). Disagreements in inclusion/exclusion were resolved by a third reviewer (A.C.). The search initially included all antivirals including broad-spectrum or nonspecific antivirals and treatments, donor polyclonal immunoglobulin, and MAbs. After the pilot screening involving the evaluation of title and abstracts, only studies involving MAb development and evaluation in an animal model were included. Articles were excluded if the full text was not available or if they were duplicates, news articles, interviews, comments, patents, or case reports.

Data analysis. After completion of article inclusion, a data-charting form was used to determine the variables to extract, and five reviewers (K.M., A.L., B.A., E.T., A.C.) independently charted the data. We extracted data on study design (e.g., aim, MAb dosages tested, prophylactic and/or therapeutic treatments evaluated, animals tested including sex, size, strain, age, lethal challenge model, inoculation routes), study results including viral epitope target, MAb in vitro characterization, and the main findings of the study. We grouped studies based on viral protein target, MAb cross-reactivity, and novel MAb configurations or delivery methods.

## 3. Results

**Figure 1:**
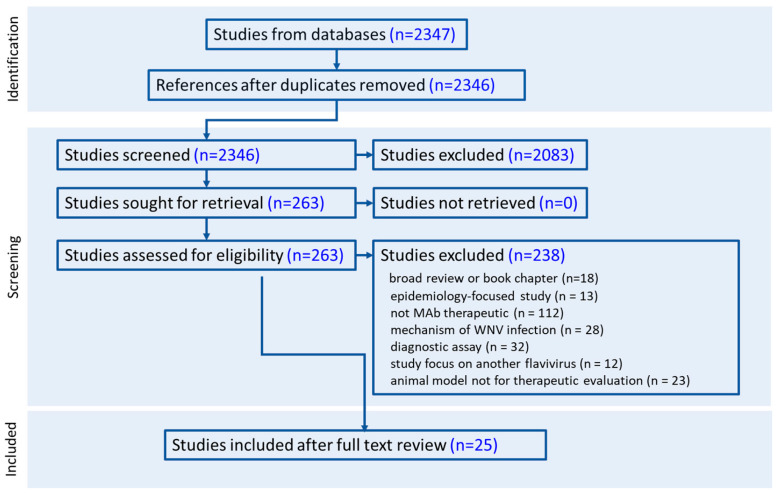
PRISMA flow diagram of the citations analyzed throughout the scoping review process.

The database search yielded 2347 papers, with one duplicate removed (Figure 1). The remaining 2346 papers were screened. Upon further evaluation of the inclusion criteria to only include those studies evaluating MAbs for their therapeutic efficacy in an animal model, 263 papers were assessed for eligibility. Of these, 238 papers were excluded from the analysis. Many papers were excluded because they were not a MAb therapeutic (*n* = 112), described mechanisms of WNV infection (*n* = 28), focused on another flavivirus (*n* = 12), did not focus on the evaluation of a therapeutic MAb (*n* = 23), focused on WNV epidemiology (*n* = 13), or were a broad review or book chapter (*n* = 18). In addition, another 32 studies detailing diagnostic approaches were excluded. A total of 25 studies were included for full-text review. These included 13 studies detailing MAbs targeting the WNV envelope (E) protein [12,13,14,15,16,17,18,19,20,21,22,23,24], three studies evaluating MAbs targeting the WNV NS1 or pre-membrane protein (prM) [25,26,27], four studies detailing the development and evaluation of MAbs specific to another flavivirus and evaluated as WNV therapeutics [28,29,30,31], and five describing novel antibody conformations or delivery methods.

### 3.1. MAbs Targeting the Envelope Protein

Several studies investigated the therapeutic potential of the anti-WNV murine MAb E16, which recognizes an epitope on the lateral ridge of DIII of the E protein (E-DIII), with high neutralizing activity [18,32] (Table 1). Oliphant et al., 2005 [18] found that E16 could protect C57BL/6-outbred mice when 500 µg was administered 48 h after lethal infection with a >90% survival rate. When administered 4 days post-infection (pi), 80% of mice survived challenge. Two murine MAbs, E24 and E34, were also shown to protect mice from lethal WNV infection. When administering 500 µg of E24 either 48 h or 4 days pi, a 90% and 50% survival rate were observed, respectively. MAb E34 was even more effective with a 100% survival rate when 500 µg of antibody was administered 48 h pi. The administration of 100 µg of murine–human chimeric (ChE16) or humanized (HmE16.1) derivatives of MAb E16 48 h after lethal infection protected 90% and 100% of mice from death, respectively (Table 1) [18]. Morrey et al., 2006 [20] found that a single intraperitoneal (IP) injection of 60 mg/kg of HmE16 administered 48 h pi resulted in 95% protection in Syrian golden hamsters. When 100 mg/kg of HmE16 was administered five days after lethal WNV infection, an 80% survival rate in hamsters was observed (Table 1) [20]. Morrey et al., 2008 [19] extended this initial study to determine whether HmE16 could prevent acute flaccid paralysis from WNV infection when the virus was directly injected into the sciatic nerve or spinal cord of hamsters. When HmE16 was administered IP two to three days pi, a significant reduction in paralysis and mortality was observed (Table 1) [19]. Zhang et al., 2009 [12] studied the potential for WNV mutations in the HmE16 epitope to confer resistance to neutralization in vivo and found that Swiss Webster mice treated prophylactically with antibody and challenged with a biologically relevant WNV dose protected 80% of mice, but virus recovered from the brain of euthanized animals contained mutations in the epitope recognized by HmE16 (Table 1). This study highlights the potential for antibody therapy to select for neutralization escape variants either by selecting for naturally occurring pre-existing variants or through prolonged exposure to antibody treatment [12]. Smeraski et al., 2011 investigated the effect that treatment with humanized E16 (using manufactured doses for human clinical trials called MGAWN1) had on preventing WNV-induced spatial memory impairment and found that 92% of mice treated with MGAWN1 had improved spatial memory compared with 56% of control mice 28 days after acute infection (Table 1) [16]. Throsby et al., 2006 described a human MAb CR4374 also recognizing an epitope on the lateral ridge of E-DIII. They found that 15 mg/kg administered 24 h before lethal WNV infection was enough to protect 100% of BALB/c mice (Table 1) [15].

Other studies investigated the therapeutic effect of MAbs targeting epitopes on domains I and II of E protein (E-DI and E-DII) that are less potent at neutralizing the virus, but may still offer some protection against lethal WNV infection (Table 1). Gould et al., 2005 [21] isolated a panel of human single-chain variable region antibody fragments (scFvs) recognizing epitopes on E-DI and E-DII of the WNV E protein and found some were 100% protective in C57BL/6 mice when given 24 h before infection. Two of these (11 and 15) protected the mice therapeutically (80% survival rate) when administered at both one and four days pi even though these MAbs were not potent neutralizers in vitro [21]. Oliphant et al., 2006 [17] investigated MAbs recognizing epitopes on DI and DII including the fusion loop in DII and hinge region of DI and DII, and found that they were able to protect C57BL/6 mice from WNV (≥75% survival rate) when administered 24 h before lethal infection; however, their efficacy was less protective than the DIII-specific MAbs described above and might cause enhanced infection based on in vitro studies with Fcγ receptor-expressing cells (Table 1) [17]. Vogt et al., 2011 [14] tested the murine–human chimeric MAb E28 recognizing an epitope on the fusion loop of DII, and found that it protected mice in vivo even though it was poorly neutralizing in vitro. Following experiments with knockout mice, the authors found that E28’s in vivo efficacy was dependent upon the Fc effector function of the antibody and required phagocytic cells, C1q, and FcγRIII (Table 1) [14]. One study investigated the combinatorial effect of two anti-WNV MAbs on the protective efficacy for WNV infection [24]. Yang et al., 2023 found that two anti-WNV E MAbs were more effective (66.7% survival rate) at treating WNND when administered together than when administered separately (20% and 40% survival rate) [24].

MAbs recognizing complex quaternary epitopes on the virion particle have been shown to be potent virus-neutralizing MAbs (Table 1). Vogt et al., 2009 characterized a WNV-specific human MAb, CR4354, recognizing an epitope across two E molecules in the virion particle [13,33]. This MAb was able to confer 100% protection in C56BL/6 mice with as little as 0.42 µg given prophylactically (Table 1) [13]. Goo et al., 2019 [22] characterized a human MAb, WNV-86, that neutralized the virus in vitro with as little as 2 ng/mL. This MAb recognizes an epitope on DII on mature virions lacking the prM protein. MAb WNV-86 protected 100% of C57BL/6 mice from lethal WNV challenge when administered 48 h pi (Table 1) [22].

### 3.2. MAbs Targeting Other Viral Proteins

Two studies examined the ability of anti-NS1 MAbs to protect mice from lethal WNV challenge (Table 2). Chung et al., 2006 [26] found that, when administered concurrently as lethal WNV challenge, anti-NS1 MAbs were able to protect 75–95% of C57BL/6 mice. When 1 mg of MAb 14NS1 was administered 7 and 14 days before lethal WNV challenge, a total of 70% or 80% of mice, respectively, survived. When MAb 14NS1 was administered therapeutically 2 or 4 days pi, 68% or 60% of mice survived (Table 2) [26]. Wessel et al., 2021 isolated human MAbs from individuals with a previous WNV infection and found that when these MAbs were administered concurrently with WNV lethal challenge in C57BL/6 mice, 43–75% of mice survived depending on the MAb used for treatment (Table 2) [25].

Only one study has been conducted on the protective efficacy of anti-prM MAbs. Calvert et al., 2010 investigated the ability of human MAbs targeting epitopes on the prM protein to protect mice from lethal WNV challenge and found that when MAbs 8G8 or 5G12 were administered 24 h before challenge, only 10 and 30% of the Swiss Webster mice, respectively, and none of the interferon receptor-deficient AG129 mice survived lethal WNV infection even though the median survival times were slightly increased (Table 2) [27].

### 3.3. Flaviviral MAbs Evaluated for Cross-Protection from WNV Challenge

Four studies investigated the protective efficacy of broadly flavivirus cross-reactive MAbs to WNV infection (Table 3). Gupta et al., 2008 [30] investigated the use of MAbs raised against the JEV E protein for protection from lethal DENV and WNV challenge. They found that when MAb ascites fluid was administered 24 h before or 48 h pi, the mice were protected from lethal WNV infection by as much as 70% or 55%, respectively, depending on the MAb tested (Table 3) [30]. Deng et al., 2011 determined that broadly cross-reactive MAb 2A10G6 recognizing a fusion loop epitope could protect 80% or 37.5% of BALB/c mice from lethal WNV infection when administered 24 h before or pi, respectively (Table 3) [31]. Ozawa et al., 2018 isolated MAbs from JEV-vaccinated individuals and found that MAb WN-83, which recognizes an epitope on both DIII and the E ectodomain, protected 50% of C57BL/6 mice when administered both 24 h before and concurrently with WNV infection (Table 3) [28]. Modhiran et al., 2021 investigated the use of murine–human chimeric anti-NS1 MAb raised against dengue virus (DENV) for protection from lethal WNV infection and found that it was not protective when administered 24 h pi (Table 3) [29].

### 3.4. Novel Antibody Conformations and Delivery Methods

None of the studies described thus far investigated the passage of MAb therapy through the BBB, an important area for preclinical MAb therapeutic research. Morrey et al., 2007 [34] investigated the use of convection-enhanced delivery (CED) directly into the pons of the brain for the treatment of WNV in hamsters with hE16 (Table 4). They found that mortality was prevented when administered 5 and 6 days pi via CED; however, hE16 was not as effective when given 8 days pi via CED. The results also highlight that a small fraction of hE16 crosses the BBB when administered peripherally. Although the authors noted that the study was not performed to demonstrate eventual use in humans, it was carried out to highlight the effectiveness of hE16 in preventing neuronal infection and injury [34].

Another challenge in using MAb therapeutics is the time and cost associated with manufacturing. One approach for addressing this issue is use of adenovirus-mediated in vivo delivery (Table 4). Pereboev et al., 2008 [35] used adenovirus vector to deliver genes of the recombinant neutralizing antibody, Fc-9E2, for the in vivo production of the antibody. This resulted in up to 83% protection of Swiss Webster mice when administered 24 h before infection and 100% protection when administered concurrently or 24 h pi [35]. Three studies investigated the use of plants to produce MAb E16 for in vivo studies (Table 4). The plant-produced Hu-E16 (pHu-E16) retained its in vitro neutralizing activity and demonstrated its ability to maintain protection to C57BL/6 mice in vivo [36]. Lai et al., 2014 [37] further optimized MAb E16 as a therapeutic by expressing it as a single-chain variant and producing it in glycoengineered plants to mimic mammalian-type N-linked glycans on the molecule. The glycoengineered E16 had better virus neutralization in vitro and was able to protect mice from lethal WNV infection than parental E16 MAb (Table 4) [37]. He et al., 2014 further engineered MAb E16 to produce variants that included a tetravalent molecule (Tetra pHu-E16) exhibiting differential binding to C1q and specific Fcγ receptors to minimize the possibility of antibody-dependent enhancement from MAb treatment, and demonstrated the ability to develop MAb E16 with bifunctional characteristics (Table 4) [38].

## 4. Discussion

The number of MAb therapeutics available has increased dramatically in the last few years, and more therapies continue to be developed each year with thousands of clinical trials and preclinical studies ongoing [39]. More than 200 therapeutic MAbs have been approved for use in the United States and European Union; of these, several are used for the treatment of viral infections including Ebola virus, SARS-CoV-2, influenza A virus, Hepatitis B virus, respiratory syncytial virus, rabies virus, human immunodeficiency virus, and cytomegalovirus infections [40]. MAb therapeutics have also been shown to be effective at preventing severe disease from viral infections in immunocompromised patients [41,42]. Together with the wide breadth of technology available for MAb therapeutic development, the use of MAbs for the treatment of WNND should be revisited.

Relevant animal models are an important aspect of the preclinical evaluation of MAb therapeutics. According to guidelines from the United States Food and Drug Administration (FDA), the pathogen should produce similar disease in the animal model, such as onset, progression, symptoms, pathology, and disease outcomes, as that observed in humans. Other factors such as the route of exposure and challenge dose should also be considered [43]. The studies included in this systematic review used inbred C57BL/6 and their immune knockout derivatives; inbred BALB/c mice; outbred Swiss mice, including Swiss–Webster, NIH–Swiss, and CD-1; and immunodeficient AG129 mouse models as well as the Syrian golden hamster model to determine the efficacy of MAb treatment for WNND. C57BL/6 mice have been well-characterized for testing innate and adaptive immune responses and accurately mimic WNV CNS infection in humans [44]. C57BL/6 mice were used in 14 of the 25 studies included in this review. The routes of inoculation included intraperitoneal, subcutaneous, and footpad methods (as a surrogate for infection by mosquito bite), and the levels of baseline survival were dependent on infectious dose and inoculation route. Additionally, the studies included in this scoping review used knockout strains of C57BL/6 mice to assess the emergence of neutralization escape variants and understand therapeutic antibodies mechanisms of protection [12,14,17]. Syrian golden hamsters also were a valuable tool in studying MAb therapeutics against WNV. Syrian golden hamsters are outbred and more genetically diverse, offering an advantage over C56BL/6 and other inbred mouse models to mimic human infection. Studies involving Syrian golden hamsters in this scoping review evaluated the efficacy of MAb E16 and its derivative, MGAWN1, for reducing mortality and CNS infection leading to severe pathology in the brain and spinal cord, and improving spatial memory impairment [16,19,20,34] (Table 5). Overall, C57BL/6 mice and Syrian golden hamsters were shown to be reliable models for MAb evaluations and should be considered for use in future research studies. Likewise, challenge routes that mimic natural infection and doses that result in the highest baseline mortality should be considered in future studies.

The major target of the MAbs evaluated was the E protein, the major glycoprotein on the surface of the virion. The E protein is responsible for virus–cell membrane attachment and fusion and is made up of three domains (DI, DII, and DIII) [45,46,47]. The lateral ridge of DIII is targeted by highly potent neutralizing antibodies that work by blocking virus–cell membrane attachment. Nine of the articles included in this review investigated the use of MAb E16, which targets DIII as a prophylactic or therapeutic antibody for the prevention and treatment of WNND. MAb E16 does not block at an entry step, but in a complex with virus, enters the cell and prevents low-pH activated fusion and egress from endosomal vesicles [48]. This MAb was highly effective in both the prophylaxis and treatment of WNND when evaluated in both C57BL/6 mice and Syrian golden hamsters, increasing survival in mice and hamsters treated with the MAb more than 12-fold and 4-fold, respectively (Table 5). The humanized version, MGAWN1, was studied in a Phase 1 human clinical trial to determine safety pharmacokinetics and immunogenicity, and the researchers found that a single infusion of the antibody up to 30 mg/kg with a half-life of 26.7 days was safe and well-tolerated among study participants [49]. A Phase 2 human clinical trial was initiated for the evaluation of MGAWN1 for the treatment of WNND; however, because of a lack of enrollment of eligible subjects, the trial was prematurely discontinued [50]. The ability to quickly identify WNND patients for enrolment in the clinical trial would have been greatly improved if a rapid point of care assay for the detection of WNV were available.

Other viral targets for MAb therapeutics include DI and DII of E protein and NS1. Three of the articles included in this review targeted DI (the central domain) and II (containing the fusion loop) of E protein. MAbs that recognize the fusion loop, such as MAb E53, bind to immature and partially mature virions in structural transition that expose cryptic epitopes available for binding by the MAb and the subsequent neutralization of the virus [51]. Other mechanisms of protection of weakly neutralizing MAbs like E28 utilize Fcγ-receptor-mediated phagocytosis and the complement pathway to reduce viral dissemination [14]. Although some of these MAbs were able to protect mice from moribundity before or pi, their efficacy was not as strong as MAbs targeting E-DIII, and was dependent on the specific MAb tested, the amount and inoculation route, and the timing of inoculation.

NS1, a glycoprotein expressed on the surface and secreted from infected cells, was described as a target in three of the articles included. NS1 is highly immunogenic, leading to the production of anti-NS1 antibodies during infection. Immunization with NS1 has demonstrated protection against flavivirus challenge [52]. Anti-NS1 MAbs may offer protection from lethal WNV challenge by binding to NS1 expressed on the infected cell surface, resulting in Fcγ receptor-mediated phagocytosis [53]. Although anti-NS1 MAbs were less protective than those MAbs targeting the E protein in these studies, with increases in survival ranging from two- to six-fold, the incorporation of NS1 MAbs into a panel of therapeutic antibodies might increase potency and prevent resistance to treatment (Table 5). The addition of NS1 to a MAb cocktail also has the advantage of reducing the possibility of antibody-dependent enhancement (ADE) of infection, a phenomenon seen in dengue viral infections in which sub-neutralizing antibodies bind to virions and shuttle virus into cells via Fc receptor interactions [54]. Future research should include evaluations of combination antibody therapies for multiple viral targets and include MAbs with different mechanisms of protection for potential increased effectiveness and a decreased possibility of viral escape.

Flavivirus infection leads to the production of both virus-specific and flavivirus cross-reactive antibody responses, with flaviviruses grouped into several sero-complexes based on the ability of the heterologous polyclonal immune serum to cross-neutralize [55]. Although most broadly cross-reactive MAbs, especially those that target the fusion loop of the E protein, are less neutralizing in vitro, some might offer potential protection from heterologous flaviviral infection. Four studies included in this review tested the ability of cross-reactive MAbs to protect mice from lethal WNV infection with varying results, ranging from no improvement in survival up to 80% survival with treatment (Table 5).

Five studies in this review investigated novel delivery platforms for MAb delivery. Given that WNV is a neurotropic virus, any therapeutic developed for the treatment of WNND will need to cross the BBB, a layer of cells that controls the passage of molecules into and out of the extracellular fluid of the central nervous system (CNS) [56]. Only one study included in this review investigated the efficacy of MAb treatment for WNND in the brain of infected hamsters [34]. Humanized E16 produced in plants maintained its ability to inhibit WNND in mice when administered after infection [36], while structurally modified derivatives developed for easier passage across the BBB maintained therapeutic efficacy [37,38]. One article investigated the use of the adenovirus-mediated gene delivery of MAb, allowing for passive immunization without the need for purified MAb preparations and a longer expression of MAb over time [35] (Table 5). Adenovirus vectors have been shown to be a safe method of gene delivery with increased CNS penetration. Zolgensma, an FDA-approved treatment for patients < 2 years of age with spinal muscular atrophy, utilizes adenovirus vector 9 (AA9) for the delivery of the survival motor neuron 1 (SMN1) gene to motor neurons in the central nervous system for the production of the SMN protein [57].

New approaches to MAb therapy and BBB delivery have been developed and could prove effective in a WNV MAb therapeutic. Delivery methods to circumvent the BBB include ultrasound with microbubbles, extracellular vesicles, and nanocarriers to aid in receptor-mediated transcytosis [58,59]. Even simple methods such as the intranasal delivery of antibodies has proven to be an effective route of administration [60]. Likewise, new approaches to engineering Fc regions of MAbs to enhance the binding of neonatal Fc receptor (FcRn), bi-specific antibodies that target transferrin receptor (TfR), and nanobodies (heavy-chain antigen-binding domains) that are smaller (12–15 KDa) allow for easier passage across the BBB [58,60,61,62].

An exhaustive literature search was conducted to include all relevant published literature that investigated the use of MAbs for WNND prophylaxis and treatment, and adherence to scoping review methods was maintained. However, there are some limitations with this scoping review. First, research conducted in private industries might not have been fully accounted for, and the search was dependent on publicly available data. Only articles published in English were included, and therefore some relevant articles may have been excluded. Another limitation is the tendency for research studies to only report treatments that had positive outcomes, and reports investigating treatments with negative or neutral outcomes may have been overlooked or not available in the literature. A meta-analysis of the data presented was not feasible due to variations in experimental design, including the MAb tested and mechanism of action, dosage, route of inoculation, animal model utilized, challenge dose, and route of inoculation for challenge. While lacking a formal analysis of preclinical data, the information consolidated here should be considered in future preclinical work.

## 5. Conclusions

The development of MAb therapeutics for the prevention or treatment of WNND occurred in the earlier part of this century; however, few studies have recently been conducted. More research into new antibody designs and delivery methods to circumvent the BBB is needed. MAb treatment for WNND has the potential to alleviate the burden of disease in the United Sates, but without tools to aid in predicting WNV activity each season and rapidly diagnose patients that may be included in clinical trials, the evaluation of MAb therapeutics in humans remains elusive. Considering the important challenges regarding WNV activity prediction and timely diagnostics, the development of a prophylactic MAb that could be administered seasonally to patients at high risk of WNND will be important. To address this, new research should focus on MAb vaccines with an increased antibody half-life that could be administered yearly and at the beginning of transmission season to high-risk patients for prolonged protection throughout the season. Alternative approaches and regulatory rules need to be reevaluated to allow quicker approval of safe and effective WNV prophylactic and therapeutic MAbs that can aid in lowering the burden of WNV disease.

## Figures and Tables

**Table 1:** Preclinical studies of anti-WNV MAbs recognizing epitopes on WNV E-protein.

Study	MAb ID	Epitope Target	Dosage/Inoculation Routes	Prophylaxis/Therapy Evaluation	Animal Model	WNV Infectious Dose/Route	Baseline Survival After WNV Infection	Outcomes
Gould, et al. 2005 [21]	recombinant human IgG1 11, 15, 73, and 95	E, DI/DII	single/SC	100 µg 24 h before infection	C57BL/6 mice (4–6 weeks)	100 PFU/IP	0%	100% survival rate
	recombinant human IgG1 11 and 15		double/SC	100 µg 24 h and 4 days pi				80% survival rate
	recombinant human IgG1 11		single/SC	250 µg 24 h pi, 500 µg 3 days pi, or 500 µg 5 days pi				100%, 100%, and 60% survival rate, respectively
Oliphant, et al., 2005 [18]	murine Mabs E16, E24, E34	E/DIII	single/IP	100 µg administered 48 h pi	C57BL/6 mice (5 weeks)	100 PFU/SC	8–20%	>90% survival rate
	murine MAbs E16, E24			500 µg administered 4 days pi				80–90% survival rate
	murine Mabs E16			2 mg administered 5 days pi				90% survival rate, and cleared WNV from brain in 68% of mice by day 9
	murine-human chimeric ChE16 IgG1			100 µg administered 48 h pi				90% survival rate
	humanized hE16.1 IgG1			100 µg administered 48 h pi				100% survival rate
Morrey, et al., 2006 [20]	hE16	E/DIII	single/IP	60 mg/kg administered 48 h pi	Syrian golden hamsters (>7 weeks of age)	0.1 mL of 106.3 TCID50/mL/SC	20–35%	95% survival rate
				100 mg/kg administered 5 days pi			37%	80% survival rate
			single/IC	50 mg/kg administered 5 days pi			22%	88% survival rate
Oliphant et al., 2006 [17]	murine MAbs E60, E53, E18, E121, E113, E31, E48, E100	E, DI/II	single/IP	400 µg of MAb 24 h before infection	C57BL/6 mice (5 weeks)	100 PFU/footpad	13%	≥75% survival rate with all MAbs, exept E100
	murine MAbs E53, E60, E31, E113			500 of MAb 48 h pi	C57BL/6 mice (5 weeks)			≥39% survival rate, 85% survival rate for E31
	murine MAbs, E53, E60, E121			400 µg of MAb 24 h before infection	Fcy receptor-deficient C57BL/6 mice (8 weeks)			≥61% survival rate
Throsby et al., 2006 [15]	recombinant human IgG1 CR4374	E, DIII	single/IP	15 mg/kg of MAb 24 h before infection	BALB/c mice (4 weeks)	20 × 9.5 TCID50/IP	0%	100% survival rate
Morrey, et al., 2008 [19]	hE16	E/DIII	single/IP	32 mg/kg administered 2-, 3-, 4-, or 5 days pi	Syrian golden hamsters (>7 weeks of age)	101.8 PFU/spinal cord	0%	100% survival rate 2 days pi, 83% survival rate 3 days pi, 29% survival rate 4 days pi, 0% survival rate 5 days pi
Duan, et al., 2009 [23]	human Fab 1	E/DIII	single/IP	200 µl Fab 1 administered 24 h before infection, concurrently, or 24 h pi	C57BL/6 mice (4–6 weeks)	103PFU/IP	0%	25% survival rate
Vogt et al., 2009 [13]	recombinant human IgG1 CR4348 and CR4354	E, DI/DII	single/IP	50 µg 24 h before infection	NIH Swiss mice (3–4 weeks)	300 PFU/IP	25%	94% survival rate with CR4354
				0.014 to 14 µg 24 h before infection	C57BL/6 mice (5 weeks)	100 PFU/SC	20%	100% survival rate in mice given ≥1.4 and ≥0.42 µg of CR4348 and CR4354, respectively
Zhang, et al., 2009 [12]	hE16	E/DIII	single/IP	100 µg administered 24 h before infection	Swiss Webster (3–4 weeks)	100 PFU WNV variants/SC	0–20%	30%, 10%, and 0% survival rate with WNV NY99 variants T332K, K307E, or WNV SA58, respectively
				100 µg administered 48 h pi	congenic RAG C57BL/6 mice (5 weeks)		0–30%	100% survival rate with WNV NY99 variants T332A, T332M, T330I, or WNV SA58 K332T 10% survival rate with WNV SA58
Smeraski, et al., 2011 [16]	MGAWN1 (hE16)	E/DIII	single/IP	32 mg/kg 4.5 days pi	Syrian golden hamsters (9–10 weeks)	104PFU/SC	56% maintained spatial memory	MGAWN1 improved survival weight, weight loss, and anorexia in WNV-infected hamsters. MGAWN1-treated infected hamsters showed a 92% retention in long-term spatial memory
Vogt, et al., 2011 [14]	murine-human chimeric IgG1 MAbs E28 and E53	E, DII-fusion loop	single/IP	40 µg 24 h before infection	C57BL/6J mice (4–5 weeks)	100 PFU/footpad	14%	60% and 43% survival rate with E28 and E53, respectively
Goo et al., 2019 [22]	human MAb WNV-86	E, DII	single/IP	100 µg 48 h pi	C57BL/6 mice (5 weeks)	100 FFU/SC	10%	100% survival rate
Yang et al., 2023 [24]	C9-G11-F3 and B2-D1-H6	E	triple/IP	5 mg/kg three consecutive days pi	C57BL/6 mice (4 weeks)	106 PFU/IP	0%	20%, 40% and 66.7% survival rate with B2-D1-H6, C9-G11-F3, and B2-D1-H6 + C9-G11-F3 combined, respectively

**Table 2:** Preclinical studies of anti-WNV MAbs recognizing epitopes on WNV NS1 or M.

Study	MAb ID	Epitope Target	Dosage/Inoculation Routes	Prophylaxis/Therapy Evaluation	Animal Model	WNV infectious Dose/Route	Baseline Survival after WNV Infection	Outcomes
Chung et al., 2006 [26]	murine MAbs 10NS1, 14NS1, 16NS1, and 17NS1	NS1	single/IP	500 µg administered concurrently with infection	C57BL/6 mice (6 weeks)	100 PFU/footpad	17%	75–95% survived rate
	murine MAb 17NS1			dose response (0.8 to 500 µg) administered concurrently with infection	C57BL/6 mice (4 weeks)			45% survival rate with 4 µg
	murine MAb 14NS1			1 mg administered 7 or 14 days before infection	C57BL/6 mice (4 weeks)			70% and 80% survival rate 7 and 14 days before infection, respectively.
	murine MAbs 14NS1, 16NS1			500 µg administered 2- or 4- days pi	C57BL/6 mice (5 weeks)		10%	68% and 47% survival rate with 14NS1 and 16NS1, respectively, 2 days pi. 60% survival rate with 14NS1 and 16NS1 4 days pi.
Calvert, et al., 2011 [27]	human MAbs 8G8, 5G12	prM	single/IP	500 µg 24 h before infection	Swiss Webster (3–4 weeks)	100 PFU/IP	6%	10–30% survival rate
					AG129 IFN-receptor deficient mice (5–8 weeks)		0%	0% survival rate
Wessel, et al., 2021 [25]	human MAbs WNV-103, WNV-95, WNV-96, WNV-97, WNV-99 WNV-100, WNV-104, WNV-117, WNV-98, WNV-113, WNV-116	NS1	single/footpad	200 µg administered concurrently with infection	C57BL/6 mice (4–5 weeks)	100 FFU/footpad	10–15%	43–75% survival rates

**Table 3:** Preclinical data for MAbs derived from vaccination with another flavivirus to determine cross-protective efficacy.

Study	MAb ID	Epitope Target	Dosage/Inoculation Routes	Prophylaxis/Therapy Evaluation	Animal Model	WNV Infectious Dose/Route	Baseline Survival After WNV Infection	Outcomes
Gupta et al., 2008 [30]	murine MAb Hx-1, Hx-2, Hs-3, Hx-5	JEV-E	single/IP	100 µl ascitic fluid administered 24 h before infection	Swiss mice (3–4 weeks)	100 LD_50_/IC	0%	45%, 70%, 0%, and 25% survival rate for Hx-1, Hx-2, Hs-3, Hx-5, respectively
				100 µl ascitic fluid administered 48 h pi				40%, 0%, 55%, and 35% survival rate for Hx-1, Hx-2, Hs-3, Hx-5, respectively
Deng, et al., 2011 [31]	murine MAb 2A10G6	DENV2 E, DII-fusion loop	single/IP	200 µg 24 h before infection	BALB/c mice (4 weeks)	40 PFU/IC	0%	80% of mice survived WNV challenge when treated prophylactically
				200 µg 24 h pi				37.5% survived challenge when treated therapeutically
Ozawa, et al., 2018 [28]	human Fab WN_11, WN_39, WN_83	WNV-E from JEV-vaccinated volunteers	double/SC	350 µg administered 24 h before and concurrently with infection	C57BL/6 mice (5 weeks)	5 × LD_50_/IP	0%	50%, 0%, and 0% survival rate with WN_83, WN_39 and WN_11, respectively
Modhiran et al., 2021 [29]	murine-human chimeric 10G5.3 IgG1	DENV-NS1	single/IP	200 µg administered 24 h pi	CD1 mice (18–19 days)	1000 FFU/IP	0%	0% survival rate

**Table 4:** Preclinical studies with novel MAb conformations and delivery methods for protection efficacy against WNV infection.

Study	MAb ID	Epitope Target	Dosage/Inoculation Routes	Prophylaxis/Therapy Evaluation	Animal Model	WNV Infectious Dose/Route	Baseline Survival After WNV Infection	Outcomes
Morrey, et al., 2007 [34]	hE16	E/DIII	single/IP	32 mg/kg administered 5- and 6- days pi	Syrian golden hamsters (>7 weeks of age)	10^7.1^ TCID50/SC	<50%	>75% and <60% survival rate at 5- and 6- days pi, respectively
			single/convection enhanced delivery (CED)	43 mg/kg administered 5 days pi		10^5.3^ TCID50/SC	25%	>75% survival rate
				31 mg/kg administered 6 days pi		10^7.1^ TCID50/SC	25%	>75% survival rate
				48 mg/kg administered 8 days after challenge			55%	<45% survival rate
Pereboev, et al., 2008 [35]	Ad/Fc-9E2	E/DIII	single/IP	10^9^ PFU 24 h before, concurrently, or 24 h pi	Swiss Webster mice (4 weeks)	200 PFU/IP	20%	83.3% survival rate when administered 24 h before infection. 100% survival rate when administered concurrently or 24 h pi
Lai, et al., 2010 [36]	pHu-E16	E/DIII	single/IP	0.001 to 10 µg administered same day as infection	C57BL/6 mice (5 weeks)	100 PFU/SC	20%	80% survival rate with 10 µg of pHu-E16
				4 to 100 µg administered 48 h pi			30%	90% and 100% survival rate with 20 and 100 µg, respectively
				50 and 500 µg administered 4 days pi			10%	70% and 90% survival with 50 and 500 µg, respectively
Lai, et al., 2014 [37]	ΔXFpE16 or ΔXFpE16scFv-CH plant-derived	E/DIII	single/IP	1 ng to 1 µg administered concurrently with infection	C57BL/6 mice (5 weeks)	100 PFU/SC	20%	100% and 70% survival rate with 100 ng of ΔXFpE16 or ΔXFpE16scFv-CH, respectively.
				500 µg administered 4 days pi			10–30%	85% survival rate with 100 µg of ΔXFpE16scFv-CH.
He, et al., 2014 [38]	pHu-E16scFv-CH^1-3^	E/DIII	single/IP	1 to 100 ng same day as infection	C57BL/6 mice (5 weeks)	100 PFU/SC	20%	40%, 60%, and 80% survival rates with 1 ng, 10 ng, 100 ng, respectively.
	pHu-E16scFv-CH^1-3^ and Tetra pHu-E16			50 µg 4 days pi				75% and 90% survival rates with pHu-E16 scFv-CH^1-3^ and Tetra pHu-E16, respectively.

**Table 5:** Summary of main findings of studies included in this scoping review.

Study	Summary of Main Findings
Gould, et al., 2005 [21]	A panel of human single-chain variable fragments (scFvs) specific to WNV-E protein was developed to determine protective efficacy in a mouse model. Five scFvs offered significant protection when administered before or after WNV infection, making them promising candidates for therapeutics. Addition of the Fc region to scFvs increased the half-lives of antibody molecules as well as their protective efficacy.
Oliphant, et al., 2005 [18]	A panel of murine MAbs against E-DIII strongly neutralized WNV and recognized epitopes on the lateral ridge of DIII. MAb E16 was effective at treating mice from lethal WNV infection when administered as a single dose 5 days pi. A single dose of a humanized version, hE16, was effective at protecting mice from lethal WNV infection. Humanization of E16 will likely increase half-life in humans, and Fc mediated functions including complement fixation and cytotoxicity improving its efficacy as a therapeutic.
Chung et al., 2006 [26]	This study investigated the therapeutic potential of a panel of 22 anti-NS1 MAbs for treatment of WNV infection. A single dose of MAb 14NS1 provided significant long-lasting protection when administered up to 14 days before infection, and 2–4 days pi. Anti-NS1 MAbs may offer protection via complement activation or other complement-independent pathways. Inclusion of anti-NS1 MAbs in MAb cocktails for therapuetics may increase potency and help to prevent emergence of treatment resistance.
Morrey, et al., 2006 [20]	Humanized E16, hE16, was evaluated as a therapeutic in Syrian golden hamster model. A single dose of hE16 protected hamsters when administered 48 h or 5 days pi and reduced viral load in the brain and spleen. HE16 was effective at treating WNV infection in a second lethal animal model, demonstrating its potential utility as a treatment for WNV infection in humans.
Oliphant et al., 2006 [17]	MAbs targeting E-DII and E-DI were evaluated for their ability to protect mice from lethal WNV infection. These MAbs were not as potent neutralizers as their E-DIII counterparts but were able to protect mice from lethal WNV infection, albeit at lower efficacy than E-DIII MAbs. When evaluated in Fc-receptor deficiently mice, the protective efficacy of E-DII and E-DI MAbs was unaffected.
Throsby et al., 2006 [15]	Human MAbs generated to WNV with phage display library constructed from peripheral blood lymphocytes of three patients with previous WNV infections were examined for their ability to protect mice from lethal challenge, with CR4374 offering the most protective efficacy. The human antibody repertoire to WNV is made up mostly of nonneutralizing or weakly neutralizing MAbs specific to E-DII, and E-DIII specific MAbs while rare, are the most potent neutralizers of WNV.
Morrey, et al., 2006 [20]	This study investigated the use of hE16 for treating WNND and found that hE16 could protect hamsters from death even when administered 5 days pi with a dose of 0.32 mg/kg. Different administration routes may improve the efficacy of hE16.
Gupta et al. 2008 [30]	The study investigated the cross-protective efficacy of anti-JEV E MAbs to WNV and DENV2. While some MAbs were able to cross-protect against WNV infection, their efficacy was limited and did not provide significant protection.
Morrey, et al., 2008 [19]	Humanized E16 was evaluated for its ability to treat WNV paralysis in hamsters when administered various times after infection. MAb hE16 was able to significantly reduce paralysis and mortality when given before the onset of paralysis (approximately 48 h pi) and lowered viral load in the spinal cord. The study demonstrates the therapeutic efficacy of hE16 in reducing paralysis and mortality in an animal model.
Pereboev, et al., 2008 [35]	This study investigated whether recombinant antibodies delivered via adenovirus vector could be used to treat WNV infection. After inoculation with Ad/Fc-9E2, recombinant antibody was detected in serum within 24 h and remained detectable up to 21 days post-inoculation. When used as a prophylactic Ad/Fc-9E2 protected mice from lethal WNV infection with a survival rate of 83.3% demonstrating a novel and effective prophylactic delivery method for WNV therapeutic MAbs.
Duan, et al., 2009 [23]	Human anti-WNV Fabs were analyzed for their ability to neutralize in vitro and protect in vivo in a mouse model. Fab1 had strong neutralizing capacity in vitro but failed to protect mice in vivo implying that therapeutic MAb efficacy relies on factors beyond neutralization.
Vogt et al., 2009 [13]	Two strongly neutralizing anti-WNV human MAbs, CR4348 and CR4354, recognize a unique epitope on the virion surface at the dimer interface in DII and hinge regions between DI and DII and inhibit viral infection at a postattachment step. Their ability to protect mice from lethal WNV infection demonstrates their utility as a therapeutic MAb that may be used in combination with anti-E-DIII MAbs to enhance therapeutic capacity.
Zhang, et al., 2009 [12]	The study investigated prophylactic and therapeutic efficacy of hE16 to protect mice from WNV challenge, and the potential for treatment to select for neutralization escape variants. MAb hE16 protected mice from wild type WNV infection, but failed to protect mice infected with variants with mutations in the epitope of hE16 at positions 307 (K307E) and 332 (T332M). WNV resistant variants developed in mice treated with hE16 and exposed to high doses of virus. While hE16 offers strong protective efficacy to WNV infection, resistance may arise, particularly in immunocompromised hosts, and combinantions of MAb targeting different epitopes may improve therapeutic efficacy.
Lai, et al., 2010 [36]	hE16 produced in plants (pHu-E16) was evaluated for its ability to treat WNND in vivo. pHU-E16 was effective at protecting mice from lethal WNV infection when administered either before or after infection, and was comparable to chimeric hE16 produced in mammalian cell culture. The study highlights the effectiveness of therapeutic MAb produced in plants that may be a more cost-effective method for production.
Calvert, et al., 2011 [27]	This study describes the limited protective efficacy of non-neutralizing WNV MAbs targeting the prM protein.
Deng, et al., 2011 [31]	This study details the characterization of flavivirus cross-reactive murine MAbs 2A10G6 recognizing an epitope in the fusion loop of E-DII. While not an effective neutralizer in vitro, the MAb did afford some protection before WNV infection in vivo.
Smeraski, et al., 2011 [16]	The study investigated the effectiveness of MAb MGAWN1 (hE16) to treat WNV-infected hamsters and assessed the long-term memory retention of surviving mice using the Morris-Water Maze (MWM) test. MGAWN1 treatment prevented cognitive impairement and improved survival in treated hamsters highlighting its potential as a therapuetic MAb for the treatment of WNV by preventing long-term cognitive decline.
Vogt, et al., 2011 [14]	Anti-E DII MAbs specific to the fusion loop were investigated for their use as therapeutic MAbs for WNV infection. While non-neutralizing in vitro, MAb E28 was able to protect mice in vivo from lethal WNV infection. This protection was dependent upon Fc-mediated mechanisms of protection.
He, et al., 2014 [38]	The study investigated the efficacy of engineering pHu-E16 into a tetravalent form (tetra pHu-E16) as a bifunctional MAb for therapeutic use. Tetra pHu-E16 and other variants purified from plants retained binding and neutralization to WNV similar to hE16, and showed enhanced protection for WNND when administered therapeutically. The study demonstrates tetra pHu-E16’s utility as a potential therapeutic MAb for WNND.
Lai, et al., 2014 [37]	This study expands on previous work (Lai et al., 2010) [36] with pE16 expression in *Nicotiana benthamiana* plants that lack specific N-glycan residues to more closely resemble the mammalian glycosylation profile. A single dose of pE16 plant derivatives protected mice from lethal WNV infection even when administered 4 days after infection. This plant production platform may be a more cost-effective way to produce antibody-based therapeutics.
Ozawa, et al., 2018 [28]	Human anti-WNV MAbs were isolated using peripheral blood lymphocytes from JEV-vaccinated individuals and were shown to neutralize in vitro and in vivo offering some protection from WNV infection in mice when administered prophlactically. The study highlights the potential for cross-reactive antibodies to offer some protection to WNV.
Goo et al., 2019 [22]	Highly neutralizing human MAb WNV-86 recognizes an epitope on E-DII in mature virions lacking prM protein and protected mice therapeutically from lethal WNV infection. Compared to hE16, WNV-86 offered more robust protection in vivo highlighting its potential as a therapeutic MAb for WNND.
Modhiran et al., 2021 [29]	A broadly cross-reactive anti-NS1 DENV MAb was evaluated for its ability to confer protection in vivo against several flavivirueses including WNV. While the MAb offered some protection for DENV and ZIKV infection, no protective efficacy to WNV was observed. Even so, the study highlights the importance of a NS1 targeted vaccine and therapeutic approach for protection.
Wessel, et al., 2021 [25]	The study describes the generation of human anti-NS1 MAbs and their protective efficacy in vivo in an Fc-dependent manner. While non-neuralizing, anti-NS1 MAbs may provide therapeutic protection for WNV infection.
Yang et al., 2023 [24]	This study describes the development of WNV MAbs and evaluation for therapeutic efficacy in mice. While two MAbs offered some protection in vivo, their efficacy significantly increased when administered together. Overall, the study demonstrates the protective efficacy of MAbs for WNV infection and reduction in neuroinflammation of treated animals.
